# Vimentin as a Multifaceted Player and Potential Therapeutic Target in Viral Infections

**DOI:** 10.3390/ijms21134675

**Published:** 2020-06-30

**Authors:** Irene Ramos, Konstantinos Stamatakis, Clara L. Oeste, Dolores Pérez-Sala

**Affiliations:** 1 Department of Neurology and Center for Advanced Research on Diagnostic Assays, Icahn School of Medicine at Mount Sinai, New York, NY 10029, USA; irene.ramos.lopez@gmail.com; 2 Centro de Biología Molecular Severo Ochoa, UAM-CSIC. Nicolás Cabrera, 1, Campus de la Universidad Autónoma de Madrid, 28049 Madrid, Spain; k.s@csic.es (K.S.); oeste@cbm.csic.es (C.L.O.); 3 Department of Structural and Chemical Biology, Centro de Investigaciones Biológicas Margarita Salas, CSIC, Ramiro de Maeztu, 9, 28040 Madrid, Spain

**Keywords:** vimentin, cell surface vimentin, SARS-CoV, vimentin–pathogen interactions, anti-vimentin autoantibodies, inflammation, intermediate filaments, posttranslational modifications, tissue damage and repair, immune response

## Abstract

Vimentin is an intermediate filament protein that plays key roles in integration of cytoskeletal functions, and therefore in basic cellular processes such as cell division and migration. Consequently, vimentin has complex implications in pathophysiology. Vimentin is required for a proper immune response, but it can also act as an autoantigen in autoimmune diseases or as a damage signal. Although vimentin is a predominantly cytoplasmic protein, it can also appear at extracellular locations, either in a secreted form or at the surface of numerous cell types, often in relation to cell activation, inflammation, injury or senescence. Cell surface targeting of vimentin appears to associate with the occurrence of certain posttranslational modifications, such as phosphorylation and/or oxidative damage. At the cell surface, vimentin can act as a receptor for bacterial and viral pathogens. Indeed, vimentin has been shown to play important roles in virus attachment and entry of severe acute respiratory syndrome-related coronavirus (SARS-CoV), dengue and encephalitis viruses, among others. Moreover, the presence of vimentin in specific virus-targeted cells and its induction by proinflammatory cytokines and tissue damage contribute to its implication in viral infection. Here, we recapitulate some of the pathophysiological implications of vimentin, including the involvement of cell surface vimentin in interaction with pathogens, with a special focus on its role as a cellular receptor or co-receptor for viruses. In addition, we provide a perspective on approaches to target vimentin, including antibodies or chemical agents that could modulate these interactions to potentially interfere with viral pathogenesis, which could be useful when multi-target antiviral strategies are needed.

## 1. Introduction

Vimentin is a type III intermediate filament cytoskeletal protein expressed mainly in cells of mesenchymal origin. Therefore, it is present in fibroblasts, endothelial cells, and cells of the immune system. Vimentin filaments form a network that extends from the nuclear periphery towards the plasma membrane of cells. Vimentin carries out or modulates a plethora of cellular functions. Once thought to be dispensable due to the viability and fertility of vimentin knockout mice, it is now recognized that vimentin plays key roles in cell architecture and dynamics. Vimentin filaments interact with and modulate the function of other cytoskeletal components, and act as integrators of cellular mechanical functions, including cell migration, adhesion and division [1,2,3,4]. Vimentin also works in organelle position and homeostasis [5,6,7], aggresome formation [6,8], and protection of the nucleus in situations of mechanical stress [9]. Furthermore, it plays important roles in cell signaling by regulating the localization and activity of several MAP kinases, lipid metabolism, and even gene expression [10].

Extensive research in vimentin-deficient animal models has unveiled its involvement in response to injury or stress, with implications in wound healing, inflammation and immune response, atherosclerosis, fibrosis or infection [11,12,13,14]. Indeed, vimentin is also involved in pathology, as exemplified by its increased levels and function in fibrosis [15,16], tumorigenic transformation and tumor cell invasiveness [17,18], as well as by its role as an autoantigen in autoimmune diseases and as a receptor for pathogens [19,20] (please see [21,22] for review).

Given its implications in pathology, vimentin has become an important drug target. Nevertheless, the approaches available to harness its participation in disease are still limited. Infections by antibiotic-resistant bacteria or by newly disseminated viruses constitute global health challenges. In spite of continued research on these topics, unexpected outbreaks of infectious diseases highlight the need for new therapeutic tools. In the fight against novel pathogens, multiple targeting and combined treatments become necessary. The purpose of this review is to investigate the abundant evidence on vimentin involvement in infection and associated damage and hypothesize about its potential interest as an additional therapeutic target against certain viral infections.

## 2. General Concepts on Vimentin Structure and Assembly

The vimentin monomer is an ~54 kDa protein with a rod shape that is constituted by four alpha-helical segments joined by linkers and flanked by disorganized head (N-terminal) and tail (C-terminal) domains [23]. Although the crystal structure of the full-length protein is not yet available, numerous works employing a great variety of structural techniques have provided valuable information on vimentin organization and assembly [24,25,26,27]. Several excellent reviews have covered this topic [10,23,28], and therefore, it will be briefly considered here. The sequence of events of vimentin assembly has been deduced from exhaustive in vitro studies. Vimentin monomers possess intrinsic structural features that drive their immediate association into parallel dimers. Tetramers would then form by the anti-parallel half-staggered dimerization of two dimers. Finally, eight tetramers assemble to give rise to what is known as a unit-length filament (ULF). These ULFs associate head to tail in the elongation process, which would be followed by a radial compaction process yielding mature filaments of approximately 12 nm of width. Whereas the head domain is essential for tetramer formation and filament assembly, the tail domain appears to modulate filament compaction and bundling [29]. Vimentin polymerizes in solutions of physiological ionic strength in vitro and is mostly polymerized in non-mitotic cells [23,30]. Nevertheless, the assembly and organization of vimentin filaments in cells have not been completely elucidated. It has been proposed that vimentin filament precursors (ULFs) are transported by molecular motors on microtubules and fuse with additional units to form short filaments or squiggles, which later anneal head to tail to form filaments [31]. Moreover, filaments can associate laterally to form bundles. In addition, vimentin polymerization differs from that of tubulin or actin in that it is non-polar (it can take place at either end of the filament), subunit exchange can occur at any point along the length of the filament, and it does not require nucleotide binding. Indeed, constant exchange between soluble and polymerized vimentin occurs in cells, allowing fast and versatile remodeling of the network in response to physiological stimuli or to various kinds of stress.

The plasticity and dynamics of the intracellular vimentin network is highly dependent on the regulation of the disassembly process, which is driven by posttranslational modifications (PTMs), mainly phosphorylation [32]. In addition, modifications occurring under oxidative stress play an important role in the reorganization of the vimentin network [6,33]. Vimentin possesses a single cysteine residue (C328) that is the target for PTMs that impact its organization in vitro and in cells [34,35]. C328 is required for vimentin network remodeling in response to oxidants and electrophiles, thus acting as a sensor for these conditions, which may have pathophysiological implications [6]. Moreover, modifications at C328 can cooperate with other PTMs, such as phosphorylation, to induce filament disassembly [35]. Interestingly, cells expressing a C328S vimentin mutant display several functional defects, which highlights the involvement of this protein in multiple cellular processes [6]. In addition to phosphorylation and oxidative modifications, vimentin is the target of numerous PTMs with diverse consequences on its organization [32]. Relevant to this review are also glycosylation [36] and proteolysis. Vimentin proteolytic cleavage, either by endogenous proteases, like caspases during apoptosis [37] or calpain during hypotonic stress [38], or by proteases from pathogens, like the Moloney mouse sarcoma virus [39] or human immunodeficiency virus (HIV) [40], can provoke drastic rearrangements of the vimentin network with implications in pathogenesis.

Therefore, vimentin can exist in different assembly states in cells, which can exert distinct actions in critical cellular processes, through mechanisms not completely elucidated.

## 3. Extracellular Vimentin

**Figure 1:**
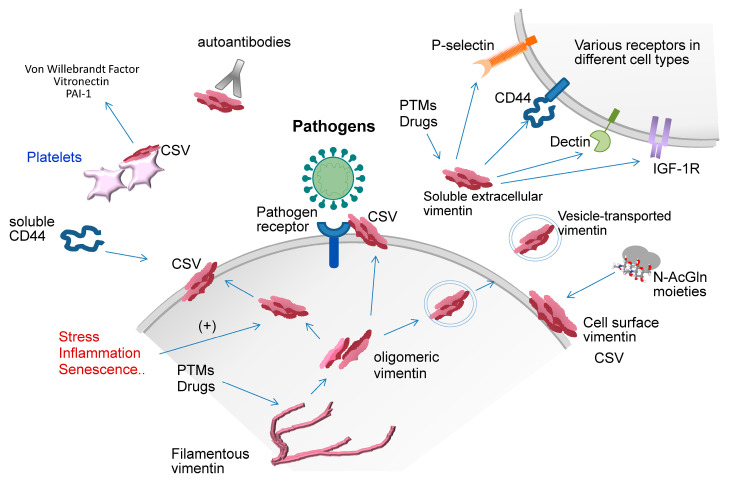
Multifaceted roles of extracellular vimentin. Vimentin, apparently in an oligomeric form, can be exposed at the surface of certain cell types, under normal and/or certain pathophysiological conditions. Additionally, vimentin can be secreted, putatively in soluble and vesicle-bound forms. For the purpose of this review, both secreted and cell-surface exposed forms will be considered extracellular vimentin. At these extracellular locations, vimentin is involved in multiple interactions and pathophysiological processes related to immune cell activation, autoimmune disease, wound healing and tissue damage, cell migration, and pathogen recognition and entry or restriction. The vimentin species participating in these diverse effects are not fully characterized and further research is needed to ascertain whether there are structure–function relationships determining these pleiotropic actions. See text for details. CSV, cell surface vimentin; IGF-1R, insulin-like growth factor 1 receptor; N-AcGln, N-acetylglucosamine; PAI-1, plasminogen activator inhibitor; PTMs, posttranslational modifications.

Vimentin is an important component of the cytoplasmic structural networks. Nevertheless, accumulating evidence situates vimentin at the cell surface or in the bloodstream, either as “soluble” forms or as a vesicle-transported protein, implying important roles in the extracellular milieu (Figure 1). Among other conditions, cell activation, apoptosis, senescence, inflammation and stress can elicit vimentin secretion [14,41].

Vimentin can be exposed at the surface or secreted by several cell types, including activated macrophages [14] and neutrophils [42], endothelial cells (HUVEC and brain microvascular endothelial cells) [43,44], and platelets [45]. Extracellular vimentin has been involved in a variety of processes, including cell–cell interaction, hemostasis, immune activation, interaction with pathogens and tissue repair (see below). Nevertheless, the mechanisms of transport to the cell surface or secretion, the structural features of extracellular vimentin, either secreted or membrane-bound, and the involvement of specific PTMs in vimentin exposure require further investigation.

Vimentin secreted by activated macrophages was reported to be phosphorylated at serine and threonine residues, and phosphatase and kinase inhibitors stimulated or inhibited secretion, respectively, thus suggesting the importance of phosphorylation in this process [14,46]. Among pathophysiological stimuli, secretion was stimulated by oxidized low-density lipoproteins [47] and TNFα and inhibited by IL-10. In this vein, Avram et al. showed that phorbol esters or cytokine activation of neutrophils stimulated the generation of tyrosyl radicals, inducing tyrosylation of various proteins, including membrane-bound extracellular vimentin [48]. In addition, upon activation, vimentin appeared as a 2D electrophoresis-resistant dimer (120 kDa apparent electrophoretical mobility) that was both tyrosylated, at undefined sites, and phosphorylated at the tail domain (T426) [48]. In turn, phosphorylation of the head domain at S72 has been shown to associate with vimentin recruitment to the cell surface upon treatment with N-acetyl-glucosamine (N-AcGln) polymers [49], thus strengthening the importance of PTM for vimentin exposure. Interestingly, extracellular exposure of the protein is frequently associated with oxidative stress [42]. Consistent with this, oxidative modifications and electrophile addition have also been reported on extracellular vimentin [33]. Vimentin exposed by CHO cells was shown to be, at least in part, reversibly oxidized [50], whereas that released by RAW264.7 macrophages stimulated with LPS has been shown to be glutathionylated [51]. In turn, vimentin exposed at the surface of human senescent fibroblasts and in plasma of aged senescence-accelerated mice was modified by addition of the electrophilic lipid malondialdehyde to C328 [41], which led these authors to propose that malondialdehyde-modified vimentin could behave as a signal for recognition and elimination of senescent cells by the immune system. In addition, since exposed vimentin is frequently oxidized, it has been proposed that it could contribute to a cascade amplifying the immune response and damage [52]. Indeed, extracellular vimentin has been reported to induce macrophage release of proinflammatory cytokines and contribute to atherosclerotic inflammation [47]. On the other hand, vimentin exposed by neutrophils undergoing spontaneous apoptosis appears to correspond to a caspase-cleaved form that retains the C-terminal domain [53].

An important modification of extracellular vimentin is citrullination, which occurs in both physiological and pathological conditions [54,55]. This is an enzymatic modification catalyzed by peptidyl arginine deiminases [56] that turns vimentin into an antigen in rheumatoid arthritis [54], but also into an antigen for anti-tumor immunity [57].

From all the above, it can be inferred that vimentin species present at the cell surface or in the extracellular medium can be structurally and functionally diverse. Indeed, the structural organization of extracellular vimentin and/or the concurrent PTMs could potentially influence its actions. Based on epitope recognition by a variety of antibodies, it has been reported that the tail domain is accessible in activated platelets [45] and on the surface of human cerebral microvascular endothelial cells [58]. Nevertheless, evidence from deletion analysis indicated that the head domain of vimentin could be involved in protein–protein interactions in the extracellular medium [44]. However, in our view, some caution needs to be exercised regarding results obtained with deletion mutants since the assembly, localization and/or interactions of constructs lacking the N-terminal or C-terminal domains could be compromised. In a recent study, Hwang and Ise have analyzed cell surface type III intermediate filament proteins and showed that they are present in multimeric complexes [59]. Moreover, they show that multimeric, but not filamentous forms, associate with lipid bilayers with high affinity, which could suggest that PTM-mediated disassembly of filamentous vimentin would be necessary for this association.

Surface-exposed or secreted vimentin can interact with several partners (Figure 1). In platelets, cell surface vimentin (CSV) has been involved in mediating adhesion at sites of injury by interaction with Von Willebrandt Factor (VWF) [45], reportedly through the tail domain. Pharmacological disruption of this interaction, either with anti-vimentin antibodies or with a VWF fragment, exerted beneficial effects in experimental models of ischemic stroke [60]. Vimentin exposed on HUVEC can interact with soluble CD44, allegedly through the head domain [44], whereas the C-terminal domain has been postulated to mediate the interaction of soluble vimentin with insulin-like growth factor 1 receptor (IGF-1R) [61]. Vimentin can also interact with Dectin-1 [62] and with pathogen proteins, which will be considered in more detail below. Importantly, since some pathogen proteins are heavily glycosylated, this binding could be favored by the lectin-like properties of cell surface vimentin. Indeed, vimentin has the ability to engage N-AcGln either on proteins in cell debris or dying cells, or on artificial matrices/polymers. This interaction has been reported to occur through coil 2 of the rod domain (residues 288 to 406), and proposed to contribute to dying cell clearance [49,63,64].

In summary, extracellular vimentin, either exposed on the cell surface or secreted, can likely exist as multiple proteoforms that exert complex and conceivably double-edged actions through interactions with diverse receptors or targets, in a context-dependent manner. A deeper understanding of these processes will be key to understand vimentin implications in pathogenesis.

## 4. Vimentin in Tissue Damage and Repair

Vimentin is both a player and a target in tissue damage and repair. Regarding its implications in infection, as it will be detailed below, pathogens and/or the associated damage can induce vimentin expression and/or surface exposure [65,66], which, in turn could hypothetically contribute to infection. Moreover, vimentin function is crucial for tissue repair, although its deregulation can lead to fibrosis [12,16]. Vimentin plays a critical role in wound healing. However, this role appears to be complex and depend on the specific tissue and time after injury. The implications of cellular vimentin in wound healing and cell migration have been the subject of several excellent reviews [2,67]. Nevertheless, the role of extracellular vimentin in tissue damage and repair is not completely characterized.

As mentioned above, vimentin is secreted by activated macrophages, helping to combat infection by acting as a proinflammatory factor, triggering the generation of oxidative metabolites and bacterial killing [14]. On the other hand, vimentin released from injured cells can bind to the surface of mesenchymal leader cells, mediating their transition to a myofibroblast phenotype and supporting wound closure [68]. In spinal cord injury, vimentin secreted by astrocytes at the injured area has been proposed to act as a neurotrophic factor, enhancing axonal growth and motor function recovery [69]. Moreover, recombinant vimentin treatment reduced acute lung injury induced by lipopolysaccharide (LPS), due, at least in part, to its binding to P-selectin, which precludes interaction with P-selectin glycoprotein ligand (PSGL-1) and the consequent interaction of leukocytes with the endothelium [70]. However, secreted vimentin can also play a pathogenic role. Levels of secreted vimentin as determined by ELISA are increased in the serum of patients with coronary artery disease, constituting an independent determinant of this condition [71]. In addition, recombinant vimentin promoted inflammatory changes in endothelial cells and macrophages, as well as inflammatory and atherogenic changes in *ApoE^−/−^* mice [71].

In turn, cell surface vimentin has relevant consequences on tissue damage and repair through interactions with some of its partners. During infection of monocytes with Mycobacterium tuberculosis, vimentin is exposed on the cell surface and acts as a ligand for NKp46 receptor on natural killer cells, inducing their activation and lysis of infected cells [72]. Moreover, monocytes are able to bind to exposed vimentin due to complement factors, IgG and fibrinogen [73,74,75]. A potential pro-repair role for surface vimentin is also supported by its recruitment to the cell surface, after phosphorylation and disassembly of the intermediate filaments, in order to bind GlcNAc-modified proteins [49] (Figure 1), and facilitate the engulfment of apoptotic cells by macrophages and mesenchymal cells [76]. Soluble and full-length CD44, the receptor of the anti-inflammatory molecule hyaluronan, bind cell surface vimentin (Figure 1) through their hyaluronan binding domains, although a mutant unable to bind hyaluronan could still bind vimentin [44]. Further studies are necessary to evaluate the effect of vimentin binding to CD44, an important receptor in lung resolution of inflammation and repair [77]. However, cell surface vimentin also has pro-coagulant properties, interacting with VWF to promote platelet adhesion [45] or VWF string formation [60]. Activated platelets exposing vimentin on their surface bind to vitronectin and active Plasminogen Activator Inhibitor 1 (PAI-1) complexes [78] (Figure 1). Vimentin stabilizes these complexes, which can inhibit epithelial repair [79] and exacerbate pulmonary fibrosis [80,81].

Vimentin is differentially expressed in proinflammatory M1 and pro-repair M2 macrophages, presenting higher levels in M2 macrophages [82], which are able to accelerate lung repair [83,84]. In the same sense, vimentin is necessary for the correct repair and remodeling of alveolar epithelial cells [85], where it is important for cell motility in repair and regeneration [67]. Thus, vimentin has important implications in lung injury and repair. Lung tissues are damaged by pathogenic infection such as viral replication inside cells, but are also damaged by the inflammatory immune response, which can be exacerbated.

Therefore, many of the documented actions of vimentin imply a necessary or beneficial role in tissue repair. Nevertheless, vimentin can also participate in pathogenic mechanisms in situations of deregulation of the immune or fibrotic responses.

## 5. Vimentin in Immune Responses

Vimentin plays a role in many key processes of the immune response. The establishment of an efficient immune response to viral or bacterial infection is critical for viral clearance and recovery. However, some viral infections are characterized by exaggerated inflammation that can cause severe immunopathology, such as acute lung injury caused by SARS-CoV-2 [86] and influenza virus infections [87]. Infection is sensed in host cells by a variety of pattern recognition receptors (PRRs), which leads to expression of cytokines and other mediators. Interestingly, vimentin has been described as a ligand for some PRRs. The nucleotide-binding oligomerization domain-containing protein 2 (NOD2) interacts with vimentin, and this interaction is required for subsequent NF-κB activation [88]. In addition, vimentin is required for assembly and activation of the NLRP3 inflammasome, which mediates the induction of pro-inflammatory cytokines associated with acute lung injury in bacterial and viral infections [12]. In turn, stimulation of the retinoic acid-inducible gene I (RIG-I), an important cytosolic PRR that detects viral RNA and mediates induction of type I and III interferon expression, leads to increased expression of vimentin [89]. Extracellular receptors also play important roles in the innate immune response to viral infections [90,91]. In this context, it has been shown that extracellular vimentin is a ligand for the PRR Dectin-1 [62], a M2 macrophage marker [83,92], the engagement of which triggers reactive oxygen species (ROS) production. Extracellular vimentin also switches the cytokine profile of LPS-activated dendritic cells by decreasing secretion of pro-inflammatory cytokines (IL-6, IL-12) and increasing IL-10 secretion, an anti-inflammatory cytokine, thereby reducing Th1 differentiation of naïve T cells [93]. Importantly, vimentin has been identified as a metabolic and functional controller of the activity of regulatory T-cells, which are critical for immune homeostasis and prevention of autoimmune and inflammatory disorders [94]. Thus, vimentin disruption enhances regulatory T-cell potency, improving their ability to suppress graft-versus-host disease [94]. A significant role was also shown for vimentin in diapedesis, i.e., crossing of leukocytes through the endothelium, where both lymphocytes and endothelial cells rely on vimentin for correct expression and distribution of adhesion-related molecules such as ICAM-1, VCAM-1 or integrin-beta1 [95]. Studies in B cells also show vimentin involvement upon antigen stimulation, for intracellular vesicles containing antigen or lysosomal associated membrane protein 1 (LAMP1) showed aberrant distribution in vimentin-deficient B cells, which are less able to present antigens, delaying in vivo antibody responses [96].

Interestingly, vimentin, usually modified by citrullination, and likely by other modifications such as oxidation and/or proteolysis, becomes an autoantigen in autoimmune disease. Indeed, these modifications could lead to the exposure of epitopes not usually accessible to the immune system and trigger an immune reaction. Anti-vimentin autoantibodies (AVA) have been detected in situations associated with tissue damage, such as chronic hemodialysis, and in transplant rejection [97] (for a recent review, see [98]). Moreover, they have a prognostic character in several pathological conditions. Therefore, the process of vimentin recognition and activation of the immune system has become an important drug target, and efforts are being devoted to develop strategies to block this process.

There are many autoimmune diseases that involve the presence of autoantibodies against vimentin. Particularly, rheumatoid arthritis (RA) patients produce antibodies against citrullinated proteins, including vimentin [19], that can be used for diagnosis. AVA can be found in immune complexes purified from synovial fluid [99] and in RA patients’ lungs [100]. Furthermore, recent data point to the role of vimentin in RA synovial fibroblasts as mediators of autoimmunity, since autophagy in these cells induces vimentin citrullination and interaction with MHC-II [55]. Circulating citrullinated vimentin is associated with disease progression and hence is also a potential biomarker of disease in ankylosing spondyloarthritis [101]. Similarly, citrullinated and matrix metalloproteinase-degraded vimentin serum levels showed high diagnostic power to differentiate Crohn’s disease from ulcerative colitis [102], and as an indicator of the response to treatment [103].

Systemic lupus erythematosus patients with kidney damage in the form of severe tubulointerstitial inflammation present high serum AVA levels. At these renal inflammatory loci, activated macrophages secrete more surface vimentin, leading to more AVA that can activate the complement system and ultimately cause tissue damage and fibrosis [104]. Recently, AVA were compared to other autoantibodies in lupus and found to be specific prognostic factors of worse response to treatment, particularly IgG AVAs [105]. Additionally, recent studies found serum antibodies against carbamoylated vimentin in lupus, an example of yet another PTM that can enhance its immunogenicity [106].

Anti-vimentin antibodies have been found in bronchoalveolar lavage fluid (BALF) from both healthy subjects and sarcoidosis patients. Nevertheless, the titers were higher in patients and the predominant reactivity was directed against the tail domain of vimentin, whereas antibodies in healthy controls were predominantly directed against the N-terminal (head) domain. Moreover, high anti-vimentin antibody levels were associated with reduced lung function. Interestingly, patients expressing HLA-DRB1*03 had a more pronounced enrichment of C-terminus-detecting AVA [107]. This HLA allotype is shared with many systemic lupus erythematosus patients, suggesting that tertiary lymphoid structures using vimentin as an antigen could appear in several chronic inflammatory diseases. In another inflammatory lung disease, namely, idiopathic pulmonary fibrosis, high circulating IgG AVA levels correlate with poor prognosis, though neither HLA allotypes nor in situ humoral immune reactions were studied in depth [108]. AVAs are also found in anti-phospholipid syndrome, where vimentin forms complexes with cardiolipin against which autoantibodies form [109].

In addition to recognition of PTM-altered vimentin, molecular mimicry-dependent cross-reactivity has been related to the development of autoimmune disease as a consequence of bacterial or viral infections [110]. In these processes, antibodies produced against pathogen antigens also recognize self-components, due to epitope similarity. For instance, antibodies recognizing streptococcal M proteins or specific proteins from herpes, measles or vaccinia viruses have been shown to recognize vimentin [111,112,113].

It is clear from the examples above that vimentin can act as a potent antigen, giving rise to autoantibodies and even in situ humoral responses (i.e., tertiary lymphoid structures). Importantly, serum vimentin levels are increased in patients with sepsis and septic shock, regulating inflammatory responses and lymphocyte apoptosis [114], which underlines its importance in immune involvement during infectious processes, as well.

## 6. Vimentin in Host-Pathogen Interactions

The involvement of vimentin in the response to pathogens presents many different features. On one hand, vimentin is an important element of the immune response and is necessary for optimal signaling in immune cells. Moreover, as stated above, vimentin acts as a ligand for pattern recognition receptors, such as Dectin-1 and the nod-like receptor protein NLRP3 inflammasome, and elicits a defense response [12,22]. However, vimentin present at the cell surface has been known for some time to act as a receptor or co-receptor for pathogens, including bacteria and viruses. Additionally, intracellular vimentin can be hijacked by pathogens leading to impairment or subversion of its functions, which then contribute to the formation of intracellular pathogen reservoirs or facilitate pathogen replication [115,116,117].

The cell or tissue distribution of vimentin expression is relevant in this context. Although endothelial and immune cells express vimentin, not all epithelial cell types, which are the target cell type in respiratory infections, for instance, express this protein, unless they have suffered injury, epithelial-mesenchymal transition or acquire an activated state [85]. Remarkably, certain lung infections, e.g., mycobacterial infections, are more frequent in patients whose lungs may be already damaged due to previous diseases, thus, likely expressing high vimentin levels as part of the process of tissue damage and repair [118]. Nevertheless, some epithelia express vimentin focally [119]. In particular, certain lung epithelial cells, including columnar and basal cells, can also express vimentin (see below) [120,121]. Numerous studies on pathogen invasion have been carried out in vitro using cancer epithelial or fibroblast-like cell lines, which express vimentin, like the Vero cell line that is frequently used in the study of viral infections. Although they may not represent ideal models, these studies have evidenced that modulation of vimentin levels by various strategies induces parallel changes in pathogen invasion [119]. In addition, certain strategies, including anti-vimentin antibodies or soluble vimentin, have been reported to reduce binding of bacterial microaggregates or viral particles to carcinoma cells [20,118].

### 6.1. Bacterial Infections

Vimentin has been involved in bacterial binding to several cell types, including endothelial, epithelial and immune cells (reviewed in [22]). Interestingly, vimentin has been reported to mediate the effect of matrix stiffness on invasion of human microvascular endothelial cells (HMEC-1) by *Lysteria monocytogenes*. In this in vitro model, vimentin was identified as a candidate surface receptor. Reducing the availability of surface vimentin, either with anti-vimentin antibodies, siRNA or chemical treatment to induce network disruption attenuated cellular infection [122]. Consistent with these findings, vimentin knockout mice have shown decreased colonization of the brain by *Listeria monocytogenes* [123], or by *E. coli* K1 invasion, thus evidencing vimentin-dependent mechanisms in bacterial meningitis [124]. *Streptococcus agalactiae* induces meningitis in newborns. Bacterial adherence to cerebral microvascular endothelial cells is facilitated by the adhesin BspC. Importantly, BspC interacts with vimentin, and vimentin-null mice are protected from *S. agalactiae* infection, suggesting that the vimentin–BspC interaction is important in the pathogenesis of meningitis [58].

Infectious agents can also modulate vimentin to facilitate infection. Agents like *Chlamydia trachomatis* or *Salmonella typhimurium* can recruit and/or remodel vimentin filaments to stabilize bacterial inclusions or to position microcolonies at a cellular location favorable for their replication [115,125]. Hijacking of vimentin functions could be mediated by pathogen-induced PTMs. Indeed, the streptococcal enzyme SpyA ADP-ribosylates vimentin at sites located in the head domain, altering its assembly [126], whereas the *Toxoplasma gondii* kinase ROP18 phosphorylates vimentin and affects its distribution [116]. Interestingly, *Chlamydia* induces O-glycosylation of vimentin to rearrange filaments for its own protection, and cells lacking the machinery to carry out this modification become resistant to *Chlamydia* infection [36].

The domains of vimentin involved in interaction with bacterial proteins appear to be variable. The *E. Coli* K1 virulence factor/invasion protein IbeA was shown to preferentially bind to a GST fusion construct of the head domain of vimentin in an overlay assay [43]. Moreover, vimentin has been reported to interact with and cooperate in the cellular uptake of C3 toxin from *Clostridium botulinum*, and this interaction appears to be mediated by the vimentin rod domain, as suggested by gel-overlay assays [127]. Interestingly, an integrin binding motif in the toxin appears to be important for binding to intact cells and to recombinant vimentin [128]. Nevertheless, further structural studies are required to characterize the interactions of vimentin with bacterial pathogens.

### 6.2. Viral Infections

**Figure 2:**
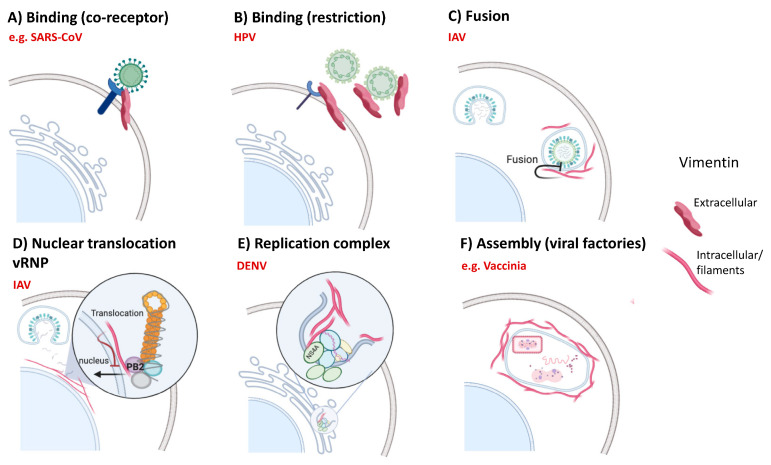
Diverse roles of vimentin during viral infections. (**A**) Vimentin has been proposed to act as a receptor or co-receptor for multiple viruses, e.g., severe acute respiratory syndrome-related coronavirus (SARS-CoV) [66]. (**B**) Extracellular vimentin (surface and soluble) restricts infection by HPV [129]. Intracellular vimentin blocks influenza A virus (IAV) infection at the fusion step [130] (**C**) and translocation of IAV viral ribonucleoproteins (vRNPs) to the nucleus [131] (**D**). (**E**) Vimentin interacts with dengue virus (DENV) NS4A at the replication complex and interferes with RNA replication [132]. (**F**) Vimentin has a role in assembly of some viruses (e.g., vaccinia virus), by supporting the formation of viral factories [133]. This illustration was created with BioRender (https://biorender.com).

Vimentin has been shown to play important roles during infection by viruses from multiple families with different types of genomes (DNA, single-stranded RNA, double-stranded RNA, etc.) and replication cycles. Remarkably, there is also great diversity in the viral cycle stages that are impacted by vimentin, with reports ranging from early stages such as binding/entry, fusion or release of virus genome to the cytosol, or later stages such as replication or assembly (Figure 2).

The SARS-CoV that caused the 2002 outbreak in China, as well as the novel SARS-CoV2 causing COVID-19, bind to their cellular receptors through their Spike (S) protein. The S proteins of these two viruses present approximately 76% amino acid identity and 87% homology (BLAST of PDB code 6VXX, SARS-CoV-2 spike glycoprotein, and PDB code 5WRG, SARS-CoV spike glycoprotein). Angiotensin converting enzyme (ACE) 2 was identified as a cellular receptor for the entry of SARS-CoV [134]. Importantly, ACE2 is also the receptor for SARS-CoV2 [135]. In addition, both viruses employ the cellular transmembrane serine protease TMPRSS2 for S protein priming [135,136], eliciting proteolytic cleavage of S after receptor binding. This cleavage leads to conformational changes in S trimers that allow for subsequent fusion of the cellular and viral membranes and facilitates virus entry [137]. In the case of SARS-CoV, there is not a strict association between ACE2 expression and entry, indicating that there might be other cellular factors important in the entry process. In this regard, a study reported an important role for vimentin in SARS-CoV virus entry through interaction with its S protein [66] (Figure 2A). Interestingly, expression of extracellular vimentin was upregulated after this interaction. It is important to note that this study was performed using the cell line Vero E6, and therefore these results remain to be confirmed in relevant human primary cells. Notably, analysis of lung samples from SARS patients has shown infection of several cell types, including pneumocytes, but also immune and endothelial cells and fibroblasts [138]. Infection of primary lung fibroblasts was also occasionally observed in an in vitro model [139]. Although interaction of vimentin with SARS-CoV2 is yet to be evaluated, a recently reported interaction map including all proteins from this virus identified potential interactions between vimentin and several viral proteins, including S protein [140]. In addition, several recent single cell RNA sequencing studies have shown that, besides pneumocytes, expression of SARS-CoV-2 entry factors, ACE2 and TMPRSS2, is particularly high in nasal epithelial goblet secretory cells and ciliated cells [141,142], in which early immunohistological studies had shown the presence of vimentin [143]. Moreover, SARS-CoV-2 infection of endothelial cells, known to express ACE2 and TMPRSS2 [144,145], has been confirmed recently [146]. However, differences in antigenicity and affinity binding to ACE2 between the S proteins from SARS-CoV and SARS-CoV-2 have been identified [147]. Therefore, further research at the protein level is needed to understand a possible role for vimentin as a receptor or co-receptor for both SARS-CoV and SARS-CoV-2.

A role for vimentin in binding and entry has been also suggested in the case of dengue virus (DENV) infections. Specifically, vimentin’s rod domain has been shown to interact with the envelope protein of the virus in endothelial cells and promote virus absorption and subsequent infection [148]. A different report showed that enterovirus 71 also interacts with vimentin at the surface of the cell, but in this case the interaction occurs between its N-terminal portion and the most external viral protein, VP1 [20]. Additional reports have shown involvement of extracellular vimentin in virus attachment and entry for Japanese encephalitis virus [149,150], porcine reproductive and respiratory syndrome virus [151], cowpea mosaic virus [152] and Chandipura virus [153]. Interestingly, binding of viral particles to vimentin at the surface of host cells had an opposite effect in the case of human papillomavirus (HPV) 16 pseudovirions [129]. Schafer et al. reported that both surface-exposed and soluble extracellular vimentin could act as a restriction factor, probably by interfering with the interaction of the virus with the attachment receptor either through direct contact or steric hindrance, limiting internalization of the virus into epithelial cells (Figure 2B).

In the case of influenza A virus (IAV), it has been shown that vimentin is necessary for the release of viral ribonucleoproteins (vRNPs) to the cytoplasm through alteration of lysosomal trafficking during entry (Figure 2C). This effect seems to be associated with impaired acidification of early endosomes in cells lacking vimentin, which is necessary for membrane fusion during early stages of infection, resulting in defective release of vRNPs to the cytoplasm [130]. Indeed, vimentin is involved in promoting an adequate distribution and function of vesicular structures along the endolysosomal pathway [5,6]. In addition, vimentin has been found to interact with the PB2 unit of the IAV polymerase [131]. This interaction occurs in the perinuclear region of the cell and results in reduced translocation of vRNPs to the nucleus (Figure 2D), where IAV genome replication takes place, as well as decreased polymerase activity. Therefore, in this case vimentin acts as a restriction factor of viral replication. Interestingly, IAV infection induces upregulation of vimentin, but also evades its antiviral function by inducing expression of miRNA-1290, which targets vimentin mRNA for degradation [131].

During DENV infection, the N-terminal region of vimentin has been shown to interact with non-structural (NS) protein 4A, a hydrophobic protein that localizes to the endoplasmic reticulum with an important role in replication. Specifically, vimentin interacts with the N-terminal cytosolic portion of NS4A in the replication complexes in the endoplasmic reticulum (Figure 2E). In this work, they also found that vimentin is reorganized during infection, which was critical for the formation of the replication complexes [132]. A similar vimentin rearrangement has been reported during Human Enterovirus Group B infection [154]. Vimentin expression in this case was shown to be important for synthesis of non-structural viral proteins, and inhibition of vimentin lead to alteration of pro-apoptotic functions of those proteins, as well as prolonged cell survival [154]. In the case of infection by foot-and-mouth disease virus, vimentin interacts with non-structural protein 3A and exerts complex effects in viral replication and yield [155].

Intracellular vimentin has also been shown to play a role during vaccinia virus infection, where vimentin filaments concentrate around foci of active viral replication in the cell, known as viral factories (Figure 2F), suggesting that they might have a part in virus assembly in this context [133]. A similar phenomenon has been reported for African swine fever virus infection, which initiated vimentin rearrangement to form a cage surrounding viral factories [156]. In this work, they found that vimentin N-terminus is phosphorylated after DNA-replication begins, which might be involved in the formation of these cage-like structures.

Additional studies have shown relevant roles for vimentin during viral infections. Vimentin has been found to interact with hepatitis C virus (HCV) core protein, which regulates its expression, impacting virus replication [157]. Vimentin is also important for efficient infection by HIV [158,159], through mechanisms that are not yet well understood. Interestingly, DeBoer et al. [158] also found that abundance of vimentin is increased in the cytoplasmic and membrane fractions in infected cell cultures. Conversely, vimentin expression has been observed to be negatively affected by virus infection in some cases. For instance, vimentin is a substrate for several viral proteases [40,160]. Infection by the Moloney mouse sarcoma virus was also found to induce vimentin cleavage resulting in a fragment lacking all or part of the C-terminal tail, as mentioned above [39].

In addition to the modulation of vimentin expression and PTMs by viruses, vimentin will also respond to host factors generated during the immune response. Indeed, vimentin expression can be regulated by proinflammatory cytokines, as well as by Type I and II interferons [161,162].

Therefore, vimentin has been shown to have important and diverse implications during virus–host interactions at the cellular level in myriad ways. The versatile nature of vimentin in terms of cellular localization, conformational arrangements and as a target for different PTMs has a critical role in these interactions. It should be noted that besides vimentin, other intermediate filament proteins can be involved in interaction with viruses, either because they participate in their entry or because they are hijacked by the virus to promote its replication cycle. Although they are not the topic of this article, information on their involvement in the pathogenesis of viral infections can be found in several review articles [163,164].

## 7. Strategies to Modulate Vimentin Function: Focus on Extracellular Vimentin-Pathogen Interactions

Given the importance of vimentin as a drug target in several diseases, and in particular its role in viral and bacterial infections, multiple strategies can be devised to interfere with its functions.

A disruption of cytoplasmic vimentin can be achieved by various means including interfering with its expression and/or distribution [165]. In addition, the functions of extracellular vimentin, either soluble or exposed on the cell surface, can be targeted by blocking antibodies or decoy peptides, as well as interfering with modifications and/or trafficking pathways leading to its exposure or release. Table 1 summarizes some of the strategies and active principles employed to target vimentin, or reported to affect its function, some of which will be discussed below. In addition, several recent reviews have considered the role of vimentin as a drug target [10,166].

### 7.1. Anti-Vimentin Antibodies

As detailed above, AVA are generated in numerous inflammatory diseases. Although in most cases they appear to play a pathogenic role, in some cases they could elicit protective effects. Indeed, both endogenously generated and manufactured anti-vimentin antibodies have been used as potential therapeutic tools, above all in cancer. In fact, antibodies against extracellular vimentin have been envisaged as anti-tumoral agents, given the important implication of vimentin in epithelial-mesenchymal transition and cell migration [167]. In particular, an antibody against cell surface vimentin, known as 86C, has been reported to bind to and cause internalization of vimentin in glioblastoma multiforme tumor cells, resulting in loss of viability, apparently mediated by increased caspase 3 activity. Moreover, beneficial effects were obtained in vivo consisting in the inhibition of tumor progression [168].

In addition, the SC5 monoclonal antibody, raised against an NK leukemic cell line, selectively recognizes vimentin located at the extracellular side of the plasma membrane in activated T cells and in viable malignant Sézary syndrome lymphocytes [171]. This antibody has been found to inhibit anti-CD3-induced proliferation and cytokine secretion of peripheral blood T cells and cell clones [184]. Furthermore, certain anti-vimentin antibodies have been shown to disrupt interactions of extracellular vimentin with its endogenous receptors, such as VWF [45,60], which could have beneficial effects.

Interestingly, anti-vimentin antibodies can also disrupt vimentin interaction with pathogen proteins, including the SARS-CoV spike protein [66], and putatively the streptococcal adhesin BspC [58], ameliorating some of the pathogenic processes in which vimentin is involved [58,60]. Therefore, these agents could hold promise for their use as therapeutic agents. Importantly, a natural monoclonal human IgG1 kappa anti-vimentin antibody derived from B lymphocytes from a patient with cervical carcinoma is undergoing clinical trials against glioma [169], in which it has shown positive results [185]. Interestingly, testing of this antibody (Pritumumab) as a strategy against COVID-19 has recently begun.

### 7.2. Chemical Agents

Several compounds have been reported to alter the organization of the vimentin network. Early works pointed to acrylamide as an agent inducing selective vimentin disruption [186]. Nevertheless, alterations in microtubules and microfilaments are also apparent in cells treated with this agent [187]. Another compound, β, β′-iminodipropionitrile was reported to reversibly disrupt the vimentin network by apparently inducing filament disassembly [188]. Nevertheless, this agent is a neurotoxin, which precludes its clinical use.

The endogenous lipid mediators known as cyclopentenone prostaglandins covalently modify vimentin, selectively targeting their single cysteine residue, and inducing a collapse of the network towards the nuclear periphery [189]. These agents and other electrophilic lipids also bind to multiple cellular targets, including the transcription factor NF-κB, which was suggested to be at the basis of their antitumoral and antiviral actions [190]. A withanolide natural product, withaferin A (WFA), was also reported to bind vimentin at the region surrounding C328, disrupting filaments in cells [191]. In addition, WFA decreased vimentin levels in several experimental systems, which could convey beneficial effects in certain pathological conditions. However, this drug also poses specificity issues, since recent works indicate that it also alters the organization of a cysteine-deficient vimentin mutant and that it may target other cellular proteins [192,193]. Other natural products bind and disrupt the vimentin network, including the garlic compound ajoene, which binds to C328 inducing thiolation and alters vimentin-dependent functions [176], and the green tea polyphenol epigallocatechin gallate that binds vimentin and inhibits its phosphorylation in cells [177]. Given the important role of C328 in vimentin function, this residue can be postulated as an interesting site to perform virtual screenings for agents with modulatory properties.

In addition, several drug screening assays have been carried out, which have resulted in the identification of compounds disrupting vimentin organization [194]. In an image-based screening, several already approved drugs were found to alter vimentin, including the statins simvastatin and mevastatin [183]. Statins, widely used to lower blood cholesterol levels, pose an interesting case. Apparently, some, but not all statins disrupt vimentin distribution and folding both in cells and in vitro, and thus have the potential to interfere with cell surface vimentin [183]. Nevertheless, other statins including fluvastatin and lovastatin could interfere with vimentin through indirect mechanisms.

Statins have long been known to induce apoptosis in a variety of cancer cell lines, in part due to their ability to inhibit the isoprenylation of proteins needed for survival [195,196]. In this process, activation of caspases can result in vimentin cleavage, since this protein is a preferential caspase substrate [37]. Indeed, fluvastatin has been reported to decrease vimentin expression through caspase cleavage, selectively in breast cancer but not in normal cells [197]. Statins are well-characterized drugs. Remarkably, many COVID-19 patients are under chronic statin treatment and, given the cardiovascular complications of the disease, continuation, and even establishment of treatment with statins is being proposed [198]. Moreover, statins, in particular simvastatin, have been reported to interfere with the entry or replication of several viruses, including HIV or Zika virus [199,200], although their efficacy in patients needs further assessment. Interestingly, a clinical trial based on the combination of simvastatin and ruxolitinib for the treatment of COVID-19 has recently begun (EudraCT Number: 2020-001405-23; https://www.clinicaltrialsregister.eu). Other clinically used drugs or nutrients with capacity to disrupt vimentin could include daunorubicin, metamizole or even paracetamol. In addition, other molecular entities found to alter vimentin distribution that have undergone clinical trials are cantharidin, carvediol and ivermectin [183]. Interestingly, ivermectin is an antiparasitic drug with broad antiviral activity in vitro that was recently reported to be highly effective against SARS-CoV-2 in cell culture models [201].

Certain chemicals may also induce vimentin cleavage by proteases that remain unidentified. This is the case of gambogic acid, a natural product with anti-inflammatory and anti-tumoral properties, which elicits vimentin cleavage in HeLa cells rendering products that are missing sequences before S51 and/or after R424 [180].

Most of the compounds listed above alter vimentin distribution. Nevertheless, whether they affect extracellular vimentin and its interactions requires further investigation. In principle, agents known to alter vimentin structure, such as simvastatin or cyclopentenone prostaglandins, could potentially affect extracellular vimentin. In addition, availability of divalent cations, including Ca^2+^, Mg^2+^ and Zn^2+^ [6,202,203], can affect the state of vimentin oligomerization and its mechanical properties. However, whether they affect extracellular vimentin has not been studied.

### 7.3. Other Strategies

Vimentin or vimentin fragments have been used as decoys to inhibit the interaction of pathogens with cell surface proteins [20,204]. Nevertheless, further studies are needed since, as stated above, vimentin can also interact with Dectin-1 on macrophages promoting ROS production [62]. In the context of viral infections, recombinant vimentin has been shown to limit cell internalization of human papilloma virus 16 pseudovirions [129] and to reduce enterovirus 71 virus infection in cellular models [20]. However, multiple interactions may exist and these strategies need to be carefully considered. Vimentin can interact with PSGL-1, which has been recently shown to act as a restriction factor for several viruses [205]. Therefore, it would be necessary to ascertain whether binding to vimentin could help or compromise this function.

Experimental strategies to modulate vimentin expression have allowed establishing correlations between cellular vimentin levels and virus infectivity. Inhibition of vimentin expression by either siRNA [20,129], or shRNA [66] have been used in cellular models, although the results obtained point to cell- and virus-dependent effects of these strategies. Since RNA interference could affect surface and intracellular vimentin, the effects can be complex, and require careful assessment.

Vimentin has also been targeted by nucleic acid aptamers, above all in anti-cancer strategies. The DNA aptamer NAS-24 was delivered inside cells or employed in nanoparticles to induce apoptosis of cancer cells [206,207]. However, to the best of our knowledge, their use against cell surface vimentin has not been documented. In turn, an RNA aptamer, P15, has been found to selectively bind vimentin on the surface of cancer cells and reduce tumorigenic behavior of pancreatic cancer cells [208]. Therefore, nucleic acid aptamers could also be tested in vimentin–pathogen interactions.

Of note, as reflected in Table 1, the specificity of the agents or approaches reported to modulate vimentin is highly variable. Indeed, many of the listed drugs or natural products available for clinical use display a relatively low specificity for vimentin. Nevertheless, although a high specificity may be required for some applications, multi-target drugs can be convenient in certain conditions. Additionally, exploring the involvement of vimentin alterations in the effects of some of these drugs would be interesting to learn about the mechanisms of their beneficial or adverse effects.

## 8. Concluding Remarks and Perspectives

Although considered at first just a building block for intermediate filaments that fulfills a scaffold function, vimentin is emerging as a versatile protein which can exist as a host of structurally and functionally diverse protein species, or proteoforms, differing in their PTMs, oligomeric state, location and interactions. Vimentin is involved in essential intracellular processes, including cell signaling, organelle positioning and function, and integration of cytoskeletal dynamics. In addition, vimentin present on the surface of cells can carry out a great variety of roles in cell–cell interactions, wound healing, migration and activation of the immune system. Importantly, cell surface vimentin has been identified as a receptor or co-receptor for pathogens. Recently, the participation of vimentin as a cellular co-receptor for SARS-CoV has been reported. Moreover, the presence of vimentin in several cell types together with other proteins involved in viral entry, such as ACE2 or TMPRSS2, grants efforts devoted to elucidate its potential role in coronavirus infections. In the context of COVID-19, identification of new pharmacological targets to design combination therapies would be desirable. Targeting vimentin in the context of viral infections could offer additional therapeutic possibilities. Some of the substances targeting vimentin, although in some instances with limited selectivity, are already in clinical use or in clinical trials. Nevertheless, given the pleiotropic character of vimentin actions, further research will be needed to establish the potentially beneficial and deleterious effects of approaches involving anti-vimentin antibodies and/or vimentin fragments. Undoubtedly, structural and functional characterization of extracellular vimentin in its various forms will be necessary for its consideration as a therapeutic target. Moreover, knowledge of the cellular mechanisms leading to the generation, trafficking and membrane targeting or secretion of extracellular vimentin would be key to be able to modulate this process.

## Figures and Tables

**Table 1:** Agents/Strategies affecting vimentin function.

Macromolecules	Clinical Use	Putative Effect	Specificity for Vimentin	References
**Expression vectors, wt and mutants/fragments**		Mimic/inhibit	Very high	[6]
**siRNAs**		Inhibit expression	High	[20,129]
**Recombinant vimentin and fragments**		Mimic/compete vimentin release or exposure	High	[44,66]
**Soluble CD44**		Compete for vimentin binding	High	[44]
**Pritumumab (anti-vimentin mAb)**	Clinic, Phase II	Membrane vimentin binding	Very high	[169,170]
**SC5 anti-vimentin mAb**		Membrane vimentin binding	Very high	[171]
**Anti-Cell surface vimentin (CSV) 86C mAb**		Membrane vimentin binding and internalization	Very high	[168]
**Anti-citrullinated Vimentin antibodies**	Diagnostic	Biomarker	Very high	[19]
**Hyaluronic acid (CTX-100)**	Phase II	Compete with vimentin for CD44	Moderate- Low	NCT00993707 *
**PEGPH20 (Pegylated Hyaluronidase)**	Phase I	Reduce hyaluronan levels	Moderate- Low	[172]
**ß-glucans (Proglucamune)**	Dietary supplement	Dectin-1 agonist	Low	[173,174]
**Dectin-1 blocking antibodies**		Block Dectin-1 signals	High	[62]
**Small Molecules**	**Clinical Use**	**Putative Effect**	**Specificity for Vimentin**	**References**
**Withaferin A**	Withania Somnifera extract (WSE; Sensoril^®^)	Reduce vimentin levels, binds region of C328, phosphorylation	Moderate	[16,175]
**Ajoene**	Garlic oil & pure studies	Disrupt vimentin network and functions, bind C328	Low	[176]
**Epigallocathechin gallate**	Dietary supplement trials	Inhibit vimentin phosphorylation	Low	[177,178,179]
**Gambogic acid**	Traditional Asian medicine	Vimentin cleavage	Low	[180,181,182]
**Simvastatin**	Clinic	Vimentin distribution; viral entry inhibition; anti-inflammatory	Low	[183] NCT04348695 *
**Cantharidin**	Phase I-IV	Vimentin distribution; antiviral	Low	[183]
**Carvedilol**	Phase I-IV	Vimentin distribution	Low	[183]
**Ivermectin**	Phase I-IV	Vimentin distribution; COVID-19 and Dengue treatment	Low	[183] NCT04343092 *

* ClinicalTrials.gov ID.

## Abbreviations

ACE	angiotensin converting enzyme
AVA	anti-vimentin autoantibodies
BALF	bronchoaveolar lavage fluid
CHO	Chinese hamster ovary
CSV	cell surface vimentin
DENV	dengue virus
HCV	hepatitis C virus
HIV	human immunodeficiency virus
HMEC	human microvascular endothelial cells
HUVEC	human umbilical endothelial cells
IAV	influenza A virus
ICAM-1	intercellular adhesion molecule 1
IGF-1R	insulin-like growth factor receptor 1
LAMP1	lysosomal-associated membrane protein 1
LPS	bacterial lipopolycaccharide
mAB	monoclonal antibody
N-AcGln	N-acetylglucosamine
NRLP3	nucleotide-binding domain, leucine-rich-containing family, pyrin domain-containing-3
NS4A	non-structural protein 4A
PAI-1	plasminogen activator inhibitor 1
PSLG-1	P-selectin glycoprotein ligand 1
PTM	posttranslational modification
PRR	pattern recognition receptor
RA	rheumatoid arthritis
RIG-I	retinoic acid-inducible gene I
ROS	reactive oxygen species
SARS-CoV	severe acute respiratory syndrome-related coronavirus
TMPRSS2	transmembrane protease, serine 2
ULF	unit-length filament
VCAM-1	vascular cell adhesion molecule 1
vRNP	viral ribonucleoprotein
VWF	Von Willebrandt Factor
WFA	withaferin A
